# Cystic echinococcosis in a domestic cat (*Felis catus*) in Italy

**DOI:** 10.1051/parasite/2018027

**Published:** 2018-05-04

**Authors:** Piero Bonelli, Gabriella Masu, Silvia Dei Giudici, Davide Pintus, Angela Peruzzu, Toni Piseddu, Cinzia Santucciu, Assunta Cossu, Nicola Demurtas, Giovanna Masala

**Affiliations:** 1 OIE Reference Laboratory for Echinococcosis, National Reference Laboratory for Cystic Echinococcosis (CeNRE), Istituto Zooprofilattico Sperimentale (IZS) of Sardinia, Via Vienna 2, 07100 Sassari Italy; 2 Exotic diseases Laboratory, Istituto Zooprofilattico Sperimentale (IZS) of Sardinia, Via Vienna 2, 07100 Sassari Italy; 3 Anatomical Pathology, Histopathology, Animal Genetics Laboratory, Istituto Zooprofilattico Sperimentale della Sardegna, Via Vienna 2, 07100 Sassari Italy; 4 Ambulatorio Dr.ssa Cossu, Via D. Millelire 33/c, 07100 Sassari Italy; 5 Freelance Veterinary Practitioner, Via Torres 36, 07100 Sassari Italy

**Keywords:** Cystic echinococcosis, molecular characterization, domestic cat, Italy

## Abstract

*Echinococcus granulosus sensu lato* is a zoonotic agent with a life cycle consisting of definitive hosts (dogs and wild carnivores), and intermediate hosts (usually ungulates). Other animals and humans may accidentally ingest eggs and contract cystic echinococcosis, acting as aberrant hosts. A 3-year-old neutered female cat was brought to a veterinary practice in Sassari (Italy) with abdominal distension. Ultrasound showed multiple intraperitoneal vesicles, which on laparotomy were found to be metacestodes of *E. granulosus*. Videos of the extraction of cysts are provided. Phylogenetic analysis based on a fragment of the cytochrome oxidase subunit 1 (*cox1*) mitochondrial gene identified the isolate as *E. granulosus* *sensu stricto* genotype G1, the most common genotype circulating in Europe and the Mediterranean basin. This is the first case report of cystic echinococcosis in domestic cats from Italy.

## Abstract (fr)

*Echinococcus granulosus* *sensu lato* est un agent zoonotique dont le cycle de vie comprend des hôtes définitifs, des chiens et des carnivores sauvages, et des hôtes intermédiaires, généralement des ongulés. Accidentellement, d’autres animaux, y compris les humains, peuvent ingérer des œufs et contracter l’échinococcose kystique, agissant comme des hôtes aberrants. Une chatte castrée de 3 ans a été amené à un cabinet vétérinaire à Sassari (Italie) avec une distension abdominale. L’échographie a montré de multiples vésicules intrapéritonéales qui, à la laparotomie, se sont révélées être des métacestodes d’*E. granulosus*. Des vidéos de l’extraction des kystes sont fournies. Une analyse phylogénétique basée sur un fragment du gène mitochondrial de la sous-unité 1 de la cytochrome oxydase (*cox1*) a identifié l’isolat comme étant *E. granulosus sensu stricto* génotype G1, le génotype le plus commun circulant en Europe et dans le bassin méditerranéen. Ceci est le premier rapport d’un cas d’échinococcose kystique chez un chat domestique en Italie.

## Introduction

Cystic echinococcosis (CE) is a parasitic disease of global importance caused by the larval stage of *Echinococcus granulosus sensu lato* (s.l.). *E. granulosus* *s.l.* belongs to the family Taeniidae and comprises different genotypes: genotypes G1-G3 (*E. granulosus sensu stricto*), G4 (*E. equinus)*, G5 (*E. ortleppi*), G6-G7, G8, G10 (*E. canadensis*) and *E. felidis* (“lion strain”) [15]. *E. granulosus sensu stricto* (*s.s*.), and in particular the G1 genotype, is responsible for the vast majority of human CE cases worldwide (88.44%) [1]. In Italy, the G1 genotype is highly represented (67%) in intermediate hosts [8,9], reaching larger percentages (89%) in some hyperendemic areas such as Sardinia, an Italian island in the Mediterranean Sea [6]. Similarly, the incidence of CE in humans is more prevalent in Insular and Southern Italy, and especially in Sardinia, where sheep farming is an important industry [4,14].

The *E. granulosus* life cycle is indirect and includes definitive and intermediate hosts. The adult tapeworm resides in the small intestine of wild and domestic carnivores. Intermediate hosts, usually ungulates, are infected by ingesting eggs released in the feces of definitive hosts. The life cycle is completed when a definitive hosts feed on offal or carcases of infected intermediate hosts. Accidentally, aberrant hosts can also ingest eggs and acquire infection [11]. Cystic echinococcosis in cats has already been reported in the past few years in South America [7], New Zealand [13], and Europe [17]. Recently, cats infected with the larval form of *E. granulosus s.s.* were found in Uruguay [2], Russia [12], and Turkey [5]. This note describes the case of a domestic cat infected with *E. granulosus* G1 genotype, confirmed by molecular analysis.

## Materials and methods

### Ethics

All procedures performed in this study, including surgical procedures in the private practice (Health Authority authorization, Prot. n. 77108/2011) were in accordance with Italian laws and the ethical standards of the *Istituto Zooprofilattico Sperimentale* (IZS) of Sardinia (D.Lgs 26/2014).

### Physical examination

A 3-year-old neutered female cat was brought to a private veterinary clinic in Sassari (Italy). Data on the animal’s history were collected by interviewing the owner. Clinical examination and ultrasound scan of the abdominal cavity were performed. The test was carried out using a Mylab30 ultrasound (Esaote, Genova, Italy) with a CA123 micro-convex and a linear LA523 multifrequency transducer.

### Exploratory laparotomy

The animal was premedicated intramuscularly with atropine sulfate (0.05 mg/kg), and 10 minutes later with ketamine (5 mg/kg). An intravenous catheter was placed to allow fluid administration. General anesthesia was mask-induced with isoflurane in oxygen and following tracheal intubation was maintained with the same anesthetic. After surgical field disinfection, the peritoneal cavity was entered by midline incision and the abdominal organs examined.

### Histopathology

Peritoneal cysts and portions of the spleen collected during the laparotomy procedure were promptly fixed in 10% neutral formalin and then embedded in paraffin following routine laboratory protocols for histopathological examination. Sections were cut serially from paraffin blocks at 4 µm and stained using hematoxylin and eosin (H&E) and modified period-acid Schiff (PAS) stain.

### Molecular analysis

Molecular identification of DNA extracted from protoscoleces fixed in 70% ethanol was performed by amplification [3] and sequencing of a short fragment of the mitochondrial cytochrome c oxidase subunit1 (*cox1*). The DNA sequence isolated from a cyst was deposited in GenBank with accession number MG722980. A Neighbor-Joining phylogenetic tree was constructed using Mega 6.0 [16] from a dataset comprising reference sequences of the different genotypes of *E. granulosus* *s.l*. retrieved from GenBank.

## Results and discussion

**Figure 1:**
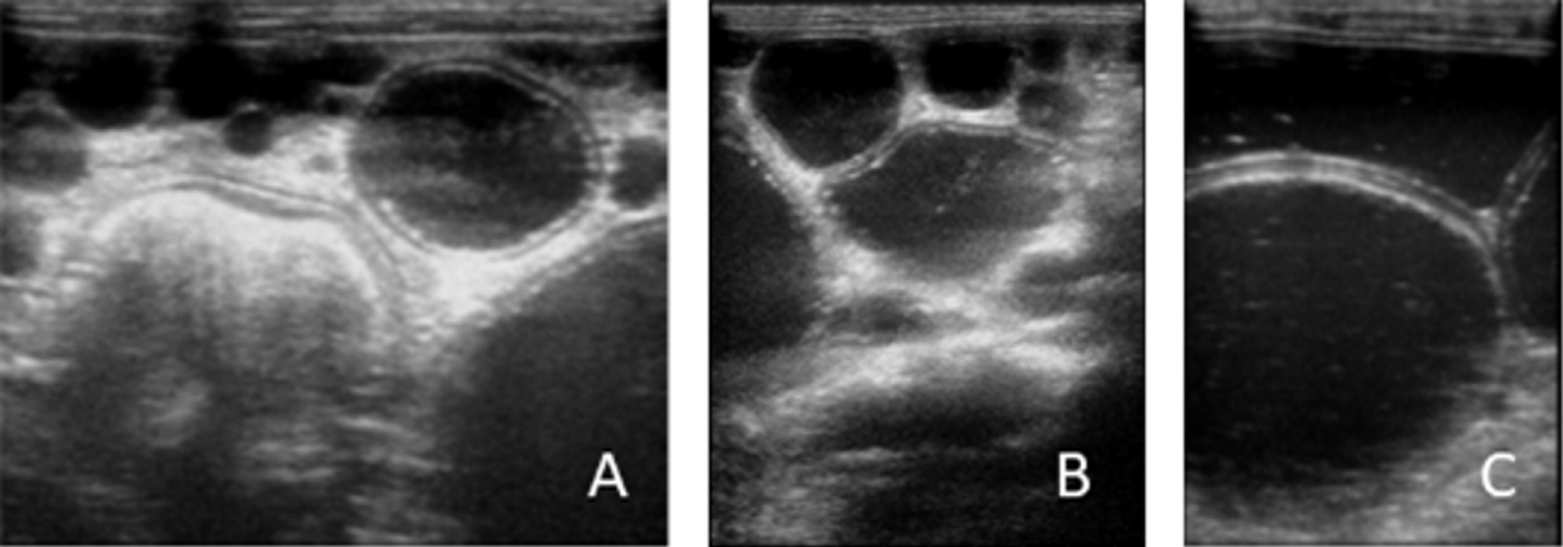
Abdominal ultrasound images. Hydatids either attached to the omentum (A) or apparently free in the peritoneal cavity (B) with anechoic content and delimited by a hyperechoic rim. Particular of a large peritoneal hydatid with evident bilaminated structure of the wall appearing as a double echogenic line separated by a hypoechogenic space (C).

Physical examination revealed abdomen enlargement, anorexia and emaciation. The history taking found that the cat was kept indoors, but could easily have access to outdoor suburban surroundings. The owner also reported that the cat’s abdomen had become more distended in the last 30 days, and that the general condition worsened a few days before consultation, when the animal showed reluctance to movement. Abdominal palpation triggered pain and revealed the presence of multiple rounded masses. Ultrasound imaging reported a voluminous splenic mass with a multiloculated structure and multiple intraperitoneal vesicles of different dimensions (Figure 1A,C) characterized by anechoic content and delimited by a hyperechoic rim. In hydatids of large dimensions, a bilaminated structure of the wall appearing as a double echogenic line separated by a hypoechogenic space was found.

**Figure 2:**
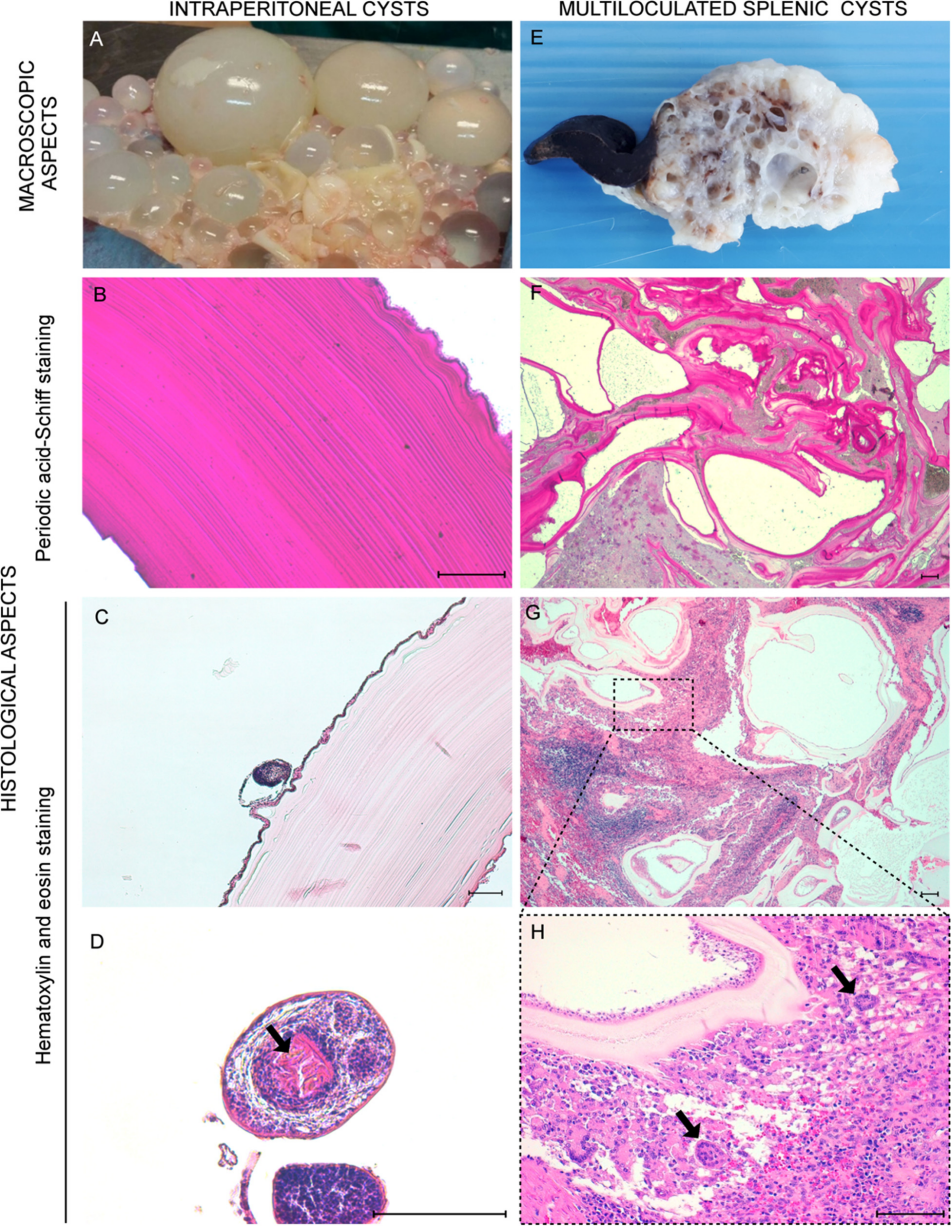
Gross and histopathological features of hydatid cysts. Free peritoneal hydatids of different dimensions extracted following laparotomy (A). Peritoneal hydatid tested by period-acid-Schiff (PAS) stain (B). Peritoneal hydatid with laminated layer and a brood capsule budding from the germinal layer (C). Protoscoleces in hydatid cavity. Notice the chain of hooks (arrows) (D). Portion of the spleen occupied by multiple cysts (E). Spleen cysts tested by period-acid-Schiff (PAS) stain (F). Spleen cysts displaying laminated, germinal and adventitial layers with inflammatory and fibrotic reaction (G); Greater magnification of the micrograph G showing the epithelioid rim around the cyst wall, giant cells (arrow) (H). Hematoxylin and eosin staining (H&E). Scale bars 100 μm.

Exploratory laparotomy showed numerous hydatids disseminated in the whole abdominal cavity that were found either free or attached to the omentum, presumably secondary hydatids as a result of the rupture of the primary splenic cyst (see Online Material, video 1-4). Peritoneal hydatids, from 5 to 40 mm in diameter, presented thin walls and contained semitransparent liquid (Figure 2A). Fertility of hydatids was determined by microscopical observation of protoscoleces, confirming that the parasite can complete full development in cats. Unfortunately, sample fixation hampered evaluation of their viability. Due to its critical clinical condition, the cat was euthanized.

Peritoneal hydatids showed the presence of an outer periodic-acid-Schiff (PAS)-positive acellular laminated layer with an inner cellular nucleated germinal layer (Figure 2B), considered to be a suggestive pattern of metacestodes of the *Echinococcus* genus [10]. Brood capsules and protoscoleces with their characteristic hooks were also detected (Figure 2C,D). A distinct encapsuled cystic mass of 65 mm in diameter protruding at one extremity of the spleen was also observed. On the cut section, it appeared sponge-like for the presence of multiloculated cysts, which were filled with yellow-grey fluid material (Figure 2E), displaying analogous histological characteristics to peritoneal hydatids except for the presence of a host-produced fibrous adventitial layer (Figure 2F,G) surrounded by inflammatory cells. Chronic granulomatous inflammation was characterized by epithelioid cells and a considerable number of multinucleated giant cells (Figure 2H).

**Figure 3:**
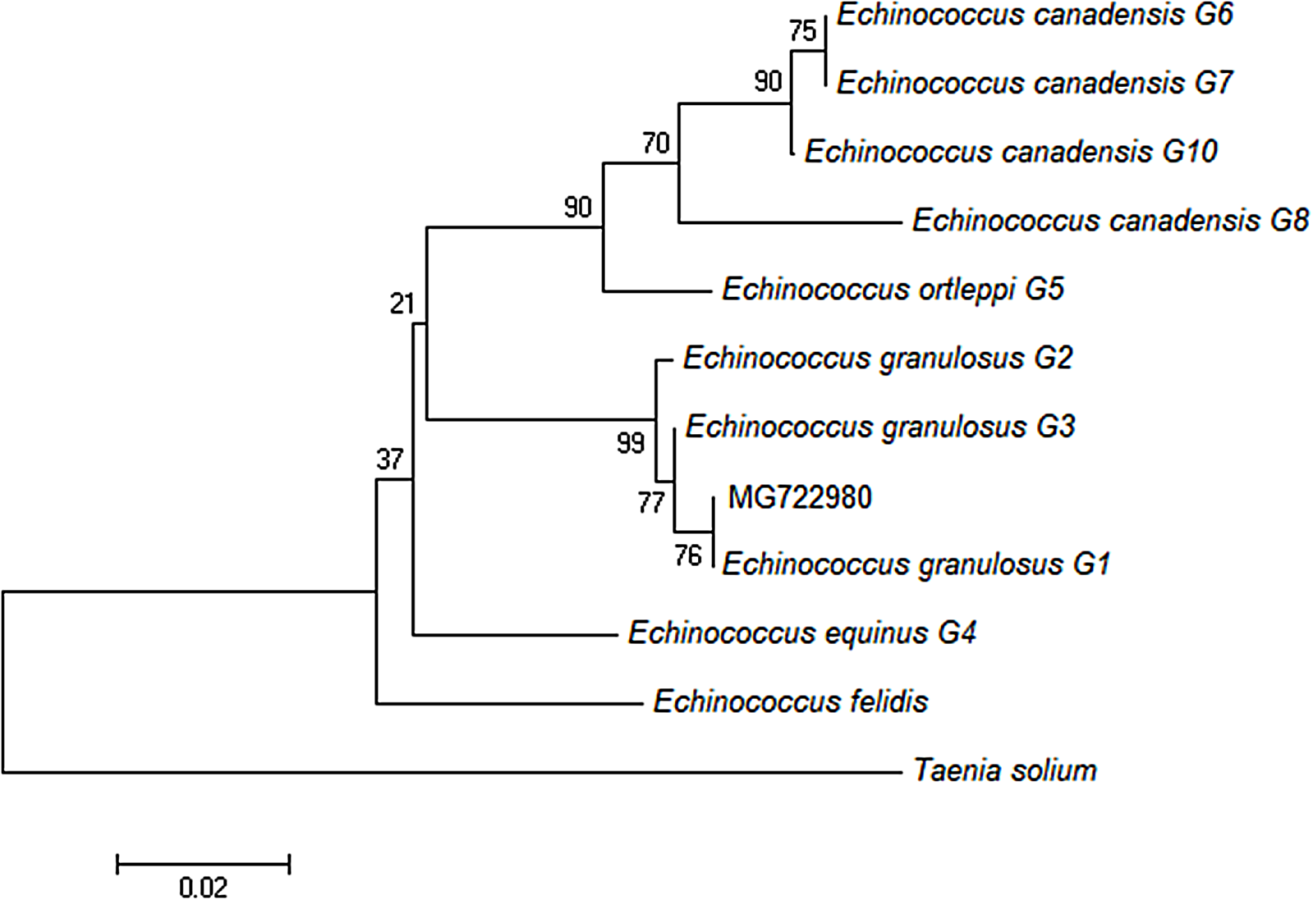
Phylogenetic tree constructed by the Neighbour-Joining method based on partial nucleotide sequences of the cytochrome oxidase subunit 1 (*cox1*) mitochondrial gene of *Echinococcus* *granulosus* *sensu lato* (*s.l*.). The phylogenetic analysis includes the sequence recovered from the domestic cat (MG722980) and GenBank sequences of *E. granulosus* *s.l*. of different genotypes: *E. granulosus sensu stricto* (*s.s*.) genotype 1 (G1) (JF513058), *E. granulosus s.s*. G2 (JF513059), *E. granulosus* *s.s*. G3 (JF513060), *E. equinus* G4 (AF346403), *E. ortleppi* G5 (AB235846), *E. canadensis* G6 (AB208063), *E. canadensis* G7 (AB235847), *E. canadensis* G8 (AB235848), *E. canadensis* G10 (AB745463). A *Taenia solium* sequence (AY211880) was used as an outgroup.

Phylogenetic analysis (Figure 3) showed that the sequence isolated from the cat belongs to the G1 cluster, differing distinctly from the other genotypes, and that it was 100% identical to the common haplotype (EG1: JF513058) previously reported as dominant in Europe [8] and in the Mediterranean basin.

## Conclusion

Cystic echinococcosis should be suspected in cats with abdominal distension and ultrasound evidence of multiple peritoneal cysts, especially in hyperendemic areas and in association with immunosuppressive diseases that may favor the development of the metacestode larva [2]. Molecular findings suggest that no specific *E. granulosus* haplotype is necessarily related to infection in domestic cats, confirming the host multitropism of the *E. granulosus* G1 genotype. This report of a clinical case of cystic echinococcosis in a domestic cat highlights the problems related to environmental contamination in urban contexts, and the associated risk for public health.

## Conflict of interest statement

The authors declare that they have no conflicts of interest in relation to this article.

## Supplementary Material

Supplemental Files 1-4, videos. Laparotomy and extraction of metacestodes of *E. granulosus* from a cat.

Video 1 Video 2 Video 3 Video 4 The Supplementary Material is available at https://www.parasite-journal.org/10.1051/parasite/2018027/olm.
